# Prediction of future gastric cancer risk using a machine learning algorithm and comprehensive medical check-up data: A case-control study

**DOI:** 10.1038/s41598-019-48769-y

**Published:** 2019-08-27

**Authors:** Junichi Taninaga, Yu Nishiyama, Kazutoshi Fujibayashi, Toshiaki Gunji, Noriko Sasabe, Kimiko Iijima, Toshio Naito

**Affiliations:** 1 Faculty of Informatics and Engineering, The University of Electro-Communications, Tokyo, Japan; 2 Department of General Medicine, School of Medicine, Juntendo University, Tokyo, Japan; 3 Center for Preventive Medicine, NTT Medical Center Tokyo, Tokyo, Japan; 4 Medical Technology Innovation Center, Juntendo University, Tokyo, Japan; 5 Clinical Research and Trial Center, Juntendo University Hospital, Tokyo, Japan

**Keywords:** Cancer, Gastrointestinal cancer

## Abstract

A comprehensive screening method using machine learning and many factors (biological characteristics, *Helicobacter pylori* infection status, endoscopic findings and blood test results), accumulated daily as data in hospitals, could improve the accuracy of screening to classify patients at high or low risk of developing gastric cancer. We used XGBoost, a classification method known for achieving numerous winning solutions in data analysis competitions, to capture nonlinear relations among many input variables and outcomes using the boosting approach to machine learning. Longitudinal and comprehensive medical check-up data were collected from 25,942 participants who underwent multiple endoscopies from 2006 to 2017 at a single facility in Japan. The participants were classified into a case group (y = 1) or a control group (y = 0) if gastric cancer was or was not detected, respectively, during a 122-month period. Among 1,431 total participants (89 cases and 1,342 controls), 1,144 (80%) were randomly selected for use in training 10 classification models; the remaining 287 (20%) were used to evaluate the models. The results showed that XGBoost outperformed logistic regression and showed the highest area under the curve value (0.899). Accumulating more data in the facility and performing further analyses including other input variables may help expand the clinical utility.

## Introduction

Gastric cancer is one of the most common cancers in the world, and Japan has one of the highest incidences^1,2^. Gastric cancer also has high mortality rates in East Asian countries^3,4^. Long-term *Helicobacter pylori* infection, pernicious anaemia and high salt intake can lead to chronic superficial gastritis, chronic atrophic gastritis and eventually intestinal epithelial metaplasia, all of which are considered risk factors for the development of gastric cancer^5–7^.

It is important to provide accurate, rapid screening for gastric cancer. If a patient is predicted as being at high risk, then (s)he can seek to undertake preventative measures in advance. Conversely, if a patient is predicted as being at low risk, then (s)he can avoid or reduce the frequency of (e.g. annually in Japan) upper gastrointestinal endoscopic examinations, which are accompanied by potential risks and high screening costs. A large-scale survey of 200,000 individuals who had been endoscopically examined reported a 0.13% adverse complication rate and a 0.004% mortality rate^8^. Therefore, endoscopic gastric cancer screening has been proposed in several subgroups of patients considered to be at high risk^9^.

While various environmental risk and host-related factors have been suggested to be associated with gastric cancer, rapid screening to classify patients as high or low risk of developing gastric cancer in the clinical setting is often provided based on a few main factors: age, familial history and the presence of *H. pylori* infection or atrophic gastritis.

Some recent studies have demonstrated that new methods such as machine learning and big data mining approaches are effective for improving screening, prediction, biomarker selection and disease diagnosis in the medical field^10–15^. We hypothesized that comprehensive screening using a combination of numerous factors accumulated every day in hospitals (e.g. biological characteristics, *H. pylori* infection status, endoscopic findings and blood test results) and a successful machine learning technique could lead to more accurate and rapid screening for gastric cancer.

One such advanced and successful machine learning method is XGBoost^16,17^. XGBoost uses multiple (hundreds of) classification and regression trees (CARTs), which can learn nonlinear relations among input variables and outcomes in a boosting ensemble manner, to capture and learn nonlinear and complex relations accurately (see the XGBoost subsection for technical details). Linear approaches such as logistic regression are not generally suitable for prediction models with complex correlations; however, multiple risk factors may jointly and nonlinearly help predict the risk of developing gastric cancer. Therefore, the purpose of the present study was to clarify the accuracy of a prediction model for the development of gastric cancer using comprehensive longitudinal data and machine learning algorithms.

## Results

We considered a classification problem regarding whether a subject would have a future risk of gastric cancer by predicting whether (s)he would be diagnosed with gastric cancer within the next 122 months. To study this, we collected longitudinal and comprehensive medical check-up data from 25,942 participants who underwent multiple endoscopies from 2006 to 2017 at a single facility in Japan (see the Methods section for details of the data collection). We classified the participants into a case group (y = 1) or a control group (y = 0) if gastric cancer was or was not detected, respectively, during the 122-month period. As a result, 1,431 participants (89 cases and 1,342 controls) were extracted. From the participants, 1,144 (80%) were randomly selected for use in training classification models, and the remaining 287 (20%) were used to evaluate the prediction accuracy of the constructed models. Classification performance was measured by receiver operating characteristic (ROC) curves and their area under the curve (AUC) values. In addition to the ROC and AUC values, the resulting accuracy, sensitivity, specificity and its confusion matrix determined by a cut-off value of 0.5 were reported. We constructed 10 classification models to address the following two research questions. Table 1 shows a list of the 10 constructed classification models (models A–J) using XGBoost and logistic regression, while incrementally adding input variables related to risk factors of gastric cancer (see the Statistical analysis subsection for details of the input variables).

**Table 1:** List of discriminative models.

Models	Classifier	Input variables
Model A	XGBoost	*H. pylori*^a^ serology testing
Model B	XGBoost	*H. pylori*^a^ serology testing and chronic atrophic gastritis
Model C	XGBoost	Variables in model B, gastric or duodenal ulcers including scars, GERD^b^ or Barrett’s oesophagus and post-gastrectomy
Model D	XGBoost	Variables in model C, sex, age and body mass index
Model E	XGBoost	Variables in model D, white blood cell counts, neutrophil ratio, lymphocyte ratio, eosinophil ratio, monocyte ratio, basophil ratio, platelet count, haemoglobin, mean corpuscular volume and haemoglobin A1c
Model F	LR^c^	The same variables as model A
Model G	LR	The same variables as model B
Model H	LR	The same variables as model C
Model I	LR	The same variables as model D
Model J	LR	The same variables as model E

^a^*Helicobacter pylori, H. pylori*.

^b^Gastroesophageal reflux disease, GERD.

^c^Logistic regression, LR.

### Increasing input variables to predict future gastric cancer

#### Questions

Long-term *H. pylori* infection and the presence of chronic atrophic gastritis are known risk factors for the future development of gastric cancer, and clinicians also often take the patient’s state into consideration. Our first question was whether only these two factors are sufficient to predict future gastric cancer. We aimed to compute the advantages that could be obtained by adding other medical check-up information in light of an advanced machine learning technique.

#### Results

**Figure 1:**
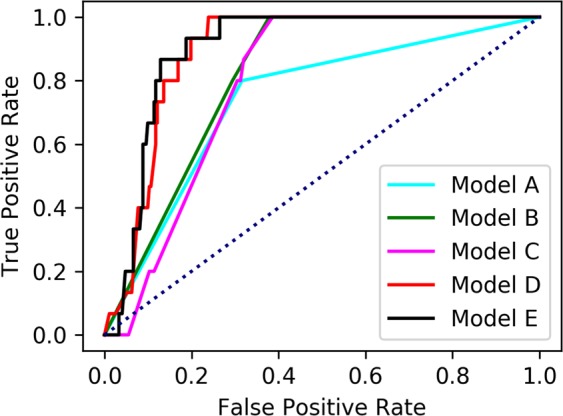
Receiver operating characteristic (ROC) curves obtained for the prediction of the development of gastric cancer.

Figure 1 shows the resulting five ROC curves for models A–E obtained using the XGBoost technique. The horizontal and vertical axes represent the false and true positive rates, respectively. Model A, which inputs solely the presence of *H. pylori* infection, is indicated by the light blue line. Model B, which inputs the presence of both *H. pylori* infection and chronic atrophic gastritis, is indicated by the green line. Model C, which added other endoscopic findings to model B, is indicated by the pink line. Model D, which added biological background factors to model C, is indicated by the red line. Finally, model E, which added blood test results to model D, is indicated by the black line. We see that the classification performance was increased by adding input variables. Especially, models D and E showed a significant increase by adding age, body mass index (BMI), and blood test results. Table 2 presents the AUC values for the cross-validation (CV) and test data corresponding to Fig. 1, along with the accuracy, sensitivity and specificity. AUC values generally increased with increasing numbers of input variables. Model E, which exploited all the information for the input variables, showed the best AUC value (0.899) for the unknown test data, with accuracy = 0.777, sensitivity = 0.933 and specificity = 0.768. In addition, the confusion matrix (true positive, false negative, false positive, true negative) for each model is shown in Supplementary Table S1. Models D and E predicted a small number of patients as false negative, which was a favourable result for a rapid screening method.

**Table 2:** Results of predicting patients at risk of developing gastric cancer.

	ROC^a^_AUC^b^ (cv^c^)	ROC_AUC (test)	Accuracy	Sensitivity	Specificity
Model A	0.736	0.742	0.690	0.800	0.684
Model B	0.792	0.815	0.641	1.000	0.621
Model C	0.823	0.790	0.690	0.867	0.680
Model D	0.858	0.885	0.763	1.000	0.750
Model E	0.874	0.899	0.777	0.933	0.768
Model F	0.736	0.742	0.948	0.000	1.000
Model G	0.792	0.815	0.948	0.000	1.000
Model H	0.822	0.799	0.634	1.000	0.614
Model I	0.853	0.880	0.941	0.000	0.993
Model J	0.862	0.874	0.885	0.600	0.901

^a^Receiver operating characteristic curve, ROC.

^b^Area under the curve, AUC.

^c^Cross-validation, CV.

In general, machine learning methods possess hyperparameters that should be carefully selected by practitioners. Different hyperparameter settings may yield different classification results. XGBoost possesses several hyperparameters (see the Hyperparameter tuning section for details) that should be optimized using training data. To optimize the hyperparameters, we took advantage of the Bayesian optimization (BO) method^18,19^, which enables the automatic selection of a favourable combination of hyperparameters using Gaussian process regression. Supplementary Figs S1 to S5 show the time course of AUC values on the CV (XGB_cv) and test data (XGB_test) as a function of XGBoost hyperparameter updates by BO. Whereas the AUC value (XGB_cv) was monotonically increasing, the AUC value (XGB_test) was not. One of the reasons for this could be the limited amount of test data. However, models A–E were observed to have a robust ranking order in terms of AUC values under several hyperparameter updates.

Lastly, Supplementary Table S2 presents importance scores for each input variable provided by XGBoost, which contribute to the prediction of future gastric cancer. In model E, HbA1c, mean corpuscular volume (MCV), lymphocyte ratio, age, BMI and post-gastrectomy were found to be more important variables. Sex was not an important variable. The automatically computed risk factors for gastric cancer were reasonable, as explained in the Discussion section below.

#### Findings

Two known risk factors, long-term *H. pylori* infection and the presence of chronic atrophic gastritis, could not accurately predict future gastric cancer. Adding other factors, biological background and blood test results, led to increased classification performance in terms of the ROC and AUC values. Important input variables automatically selected by XGBoost agreed with previous reports, as described in the Discussion section.

### Nonlinear machine learning versus linear logistic regression

#### Questions

Our second question was whether an advanced and successful nonlinear machine learning method (XGBoost) could be effective for predicting the future gastric cancer compared with a traditional linear method (logistic regression). We aimed to compute the advantages that could be obtained using XGBoost compared with linear logistic regression.

#### Results

We compared the nonlinear machine learning technique (XGBoost) with the linear method (logistic regression) as a baseline. In Table 2, models F–J show the results of linear logistic regression, which used the same input variables as XGBoost (Table 1). We found that XGBoost outperformed logistic regression when the input variables were increased (models D and I, models E and J). Whereas models A, B, C, F, G and H input only binary variables, models D, E, I and J input many continuous variables. XGBoost was able to capture the nonlinear relations between the outcome and many input variables by learning, and showed superior performance compared with traditional logistic regression. Supplementary Figs S1 to S5 show a comparison between XGBoost and logistic regression in terms of the time course of AUC values under hyperparameter updates by BO. Although XGBoost and logistic regression showed almost the same results in a few input variables (models A, B, C, F, G and H), they showed significant differences when increasing the number of input variables (models D, E, I and J).

#### Findings

In the task of predicting future gastric cancer risk, the relation between the outcome and many existing clinical markers (Table 1) was nonlinear; thus, by capturing the nonlinearity, XGBoost achieved higher performance than linear logistic regression. Increasing the number of input factors related to gastric cancer and utilizing advanced nonlinear machine learning methods appears to be effective to predict future gastric cancer risk accurately.

## Discussion

Four gastric cancer screening methods are generally performed: upper gastrointestinal series, *H. pylori* serology, serum pepsinogen test and endoscopy. It has been reported that a combination of anti-*H. pylori* antibody and serum pepsinogen can accurately predict the development of gastric cancer^20^, and in Japan, gastric cancer screening is actually performed using this combination. In a previous review, Kim *et al*.^21^ suggested that gastric cancer screening in the U.S. should be stratified by region, age, family history of gastric cancer, *H. pylori* infection and gastric conditions (atrophic gastritis/intestinal metaplasia). Comprehensive assessments of multiple risk factors may contribute to the improved accuracy of gastric cancer screening, and previous reports have suggested other factors that may be associated with the risk of developing gastric cancer, such as diabetes and obesity^22,23^. Pernicious anaemia has also been suggested to be associated with the presence of gastric cancer^24^. In addition, differential leukocyte count, including lymphocyte count, may be useful in predicting prognosis or the presence of gastric cancer^25,26^. The risk of gastric cancer may increase after gastric surgery^27^. In our comprehensive prediction models, especially model E, the indicators related to these previous reports—HbA1c, MCV, lymphocyte ratio, age, BMI and post-gastrectomy—contributed to our calculations; this resulted in higher accuracy compared with prediction model B, which used merely information regarding *H. pylori* infection and the presence of atrophic gastritis. This suggests that calculations using comprehensive data with an advanced nonlinear machine learning method improve prediction accuracy.

Our results may provide information for classifying “high-risk patients” who should be recommended for frequent endoscopic screening for gastric cancer, and “low-risk patients” who should not. Few studies have been conducted on the optimal intervals for endoscopic gastric cancer screening, and no guidelines currently exist. Gastric cancer screening is recommended every 1–2 years for high-risk patients in many countries^21^. On the other hand, in their review, Kim *et al*.^21^ recommend endoscopic gastric cancer screening every 3–5 years for low-risk patients in the U.S. With reference to these previous reports, if an accurate screening test is obtained after expanding and improving the present study, we may recommend gastric cancer screening every 1–2 years for high-risk patients and every 3–5 years for low-risk patients.

We defined patients for whom no gastric cancer could be detected for 122 months or longer as the control group. We set 122 months as a cut-off because it is the longest period that gastric cancer could be detected in the case group. It is generally thought that cancer initiation begins about 20 years before detection. Thus, the control group may have included patients who have the potential to develop gastric cancer in the future. The control group in our study did not comprise “patients who will not develop gastric cancer”, but rather “patients in whom gastric cancer will not be detected within a few years”.

Our research had several limitations. First, in our survey, we did not use information obtained from questionnaires regarding the participants’ dietary habits. It is widely known that long-term consumption of a high-salt diet is a risk factor for gastric cancer; however, our questionnaire did not collect information on salt consumption. In addition, various other environmental risk factors (e.g. smoking) and host-related factors (e.g. blood type) have been suggested to be associated with gastric cancer. Although such data were not available this time, it seems that with more related variables included, the model performance would increase.

Second, incidentally, some patients without *H. pylori* infection or atrophic gastritis were classified into the case group; the possible reasons for this are described below. It is widely known that patients in the end stage of chronic atrophic gastritis can show conversion from *H. pylori*-positive to -negative. The case group also included patients who received therapy for *H. pylori* eradication. Because our study used the initial examination data, patients with early gastric findings that were not identified may have been included in the case group.

Third, it remains unclear whether the results from our prediction can be generalized to other populations. Our predictions were calculated based on data from 1,431 patients (89 cases and 1,342 controls). It takes approximately 10 years to obtain one (control) sample for the long-term (122 months) prediction of the development of gastric cancer. We are concerned that the amount of data collected is insufficient to generalize our results. Therefore, collaborative multicentre research would be helpful.

Finally, in our study, almost all of the endoscopic findings, including part of the gastric cancer diagnoses, were diagnosed based on macroscopic findings by endoscopists; therefore, these endoscopic findings may include errors and biases. Mechanical diagnoses of stomach findings (such as the deep learning method^28^ using upper gastrointestinal endoscopic images) may be able to help avoid human errors and biases. Combining deep-learning-based diagnoses and comprehensive screening methods as in the present study is planned for our future research.

## Methods

### Study design and population

This study was conducted as a retrospective, observational, single-centre study. Most of the participants were aged between 40–60 years, and were primarily healthy clerical staff and volunteers from among the employees of the Nippon Telegraph and Telephone Corporation (NTT) and their family members. The study data were obtained from a comprehensive, periodic health check-up program carried out at the Center for Preventive Medicine, NTT Medical Center Tokyo, from May 2006 to November 2017. In Japan, the Occupational Health and Safety Law requires employers to provide annual health check-ups to ensure the health of their employees. To comply with this law, the Center for Preventive Medicine has been contracted by NTT to provide periodic medical examinations to their employees. This program includes a comprehensive periodic medical examination and many more services than required by law; the data used in this study were collected at the Center for Preventive Medicine as part of this general health check-up program. We did not specifically intend to collect new data for this study, so we extracted the participants’ past clinical data from the institution’s database. The research plan was described on the websites of both our facility and the Center for Preventive Medicine. It was announced that participants could withdraw from the study at any time without negative consequences. As this was a retrospective study, the need for informed consent was waived by the review board. The study protocol was approved by the ethics review board of Juntendo University (No. 2018148) and the institutional ethics committee at the Center for Preventive Medicine (No. 18-106). All procedures were performed in accordance with the relevant guidelines and regulations.

### Data collection

Each participant’s weight and height were measured after they removed their shoes and heavy clothing. BMI was calculated as weight in kilograms divided by height in metres squared (kg/m^2^). Serum samples were collected from each participant after overnight fasting and immediately subjected to biochemical analysis. The Japan Diabetes Society (JDS) HbA1c values were converted to National Glycohemoglobin Standardization Program values using the formula developed by the JDS: HbA_1c_ = [HbA_1c_(JDS)(%) × 1.02 + 0.25(%)]^29^. Screening using esophagogastroduodenoscopy was performed by endoscopy specialists belonging to the NTT Medical Center Tokyo. A diagnosis of gastric cancer was given when gastric cancer was strongly suspected by the endoscopic findings, or when adenocarcinoma was identified in biopsy specimens. Diagnosis of *H. pylori* infection was based on the detection of *H. pylori*-specific antibodies in serum. *H. pylori*-specific immunoglobulin G antibody titres were measured using a commercially available enzyme-linked immunosorbent assay (ELISA) according to the manufacturer’s instructions (Eiken Chem Corp., Tokyo, Japan). Participants with antibody titres 10 U/mL or higher were considered seropositive for *H. pylori* infection^30^. The sensitivity and specificity of the assay for *H. pylori* infection have been reported as follows: sensitivity 88% and <95% and specificity 84% and <95%, respectively^31,32^. Our investigation focused on 25,942 patients who underwent upper gastrointestinal endoscopy more than once between May 2006 and November 2017. Supplementary Table S3 shows the distribution of patients by the number of esophagogastroduodenoscopies obtained during the examination period. Overall, 85 patients underwent esophagogastroduodenoscopies twice, and one patient 14 times. During that period, 89 patients were diagnosed with gastric cancer, with 76 being identified as having adenocarcinoma based on biopsy specimens, and 13 strongly suspected of having gastric cancer based on endoscopic findings. The maximum examination period until the development of gastric cancer identified in the 89 patients was 122 months.

### Statistical analysis

**Figure 2:**
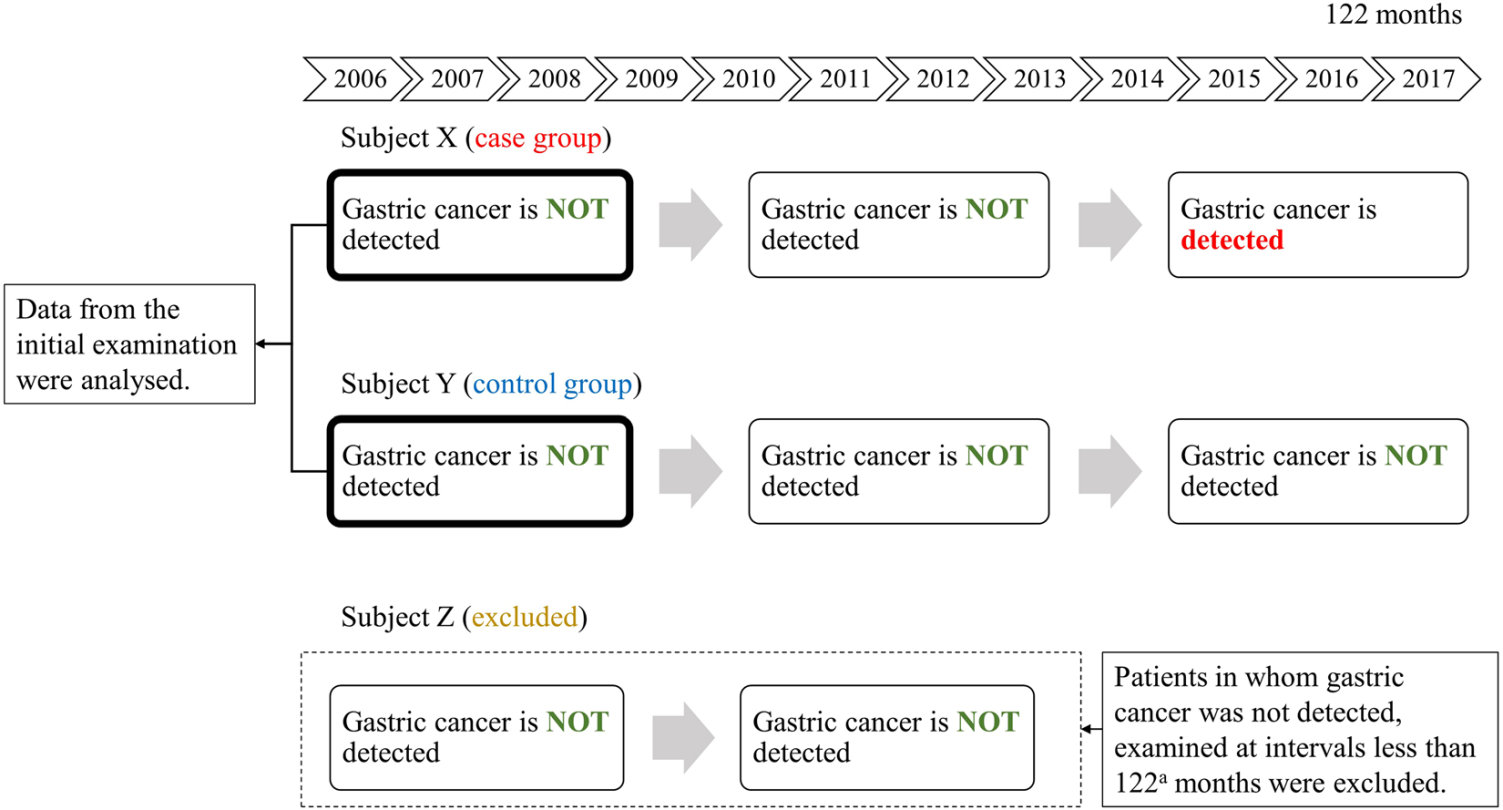
Illustration of patients with or without detected gastric cancer. (**a**) 122 months was used as the cut-off because it was the longest period that gastric cancer could be detected in the case group.

Data from each participant’s first health check-up were used to predict whether (s)he would later be diagnosed with gastric cancer during the examination period. To apply a supervised machine learning method such as XGBoost, a data label (case: y = 1, control: y = 0) is required for each participant. We defined the case group (y = 1) as patients with detected gastric cancer within 122 months (89 patients), and the control group (y = 0) as the negation: patients with no detected gastric cancer at 122 months or longer (1,342 patients). The participants could be classified into three subject categories: X, Y and Z. Subject X was for those in whom gastric cancer was detected in the second or subsequent endoscopic examinations (case group). Subject Y was for those in whom gastric cancer was not detected by endoscopy multiple times at more than or equal to 122 months (control group). Subject Z was for those in whom no gastric cancer was detected; those examined at intervals less than 122 months were excluded. Figure 2 illustrates this classification.

**Figure 3:**
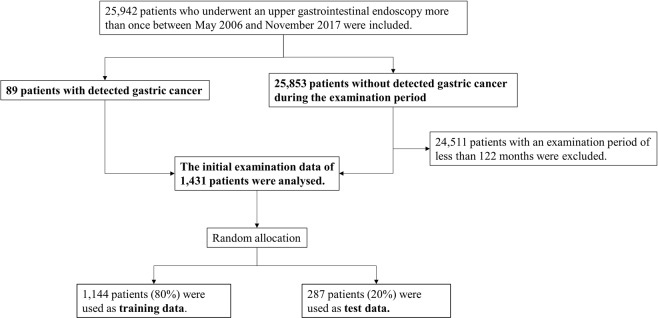
Flowchart showing the inclusion and exclusion procedures in the present study.

Table 3 shows the summary statistics of the analysed initial medical check-up data of the case and control groups. The patients in the case group were older and showed a higher prevalence of *H. pylori* infection, upper gastrointestinal ulcer and chronic atrophic gastritis. In the serologic examination, the case group had higher MCV and HbA1c values. Figure 3 shows the inclusion and exclusion procedures in our analysis. Overall, 24,511 patients with a follow-up period of less than 122 months were excluded. Finally, the initial examination data from 1,431 patients were analysed. We trained classification models by using randomly selected training data (1,144 patients [80%]), and evaluated the prediction accuracy on the remaining test data (287 patients [20%]). To predict the future risk of gastric cancer, we developed 10 classification models with different input variables (A–J), as shown in Table 1. Model A inputs information regarding *H. pylori* infection as a leading cause of gastric cancer^4,33^. Model B additionally inputs the presence of atrophic gastritis, one of the main risk factors for gastric cancer^34,35^. Model C additionally inputs other endoscopic findings considered to be risk factors for gastric cancer, including gastric ulcer (scar), duodenal ulcer (scar) and gastrectomy^36–38^. Model D additionally inputs biological characteristics such as sex, age and BMI, as obesity has been reported to be a risk factor for gastric cancer^22^. Model E additionally inputs blood test variables (complete blood count and HbA1c), as pernicious anaemia may be associated with an increased risk of gastric cancer^24,39^. In addition, the possibility that diabetes is associated with gastric cancer has been reported^23,40^. Compared with XGBoost, models F–J were calculated using the same variables by logistic regression.

**Table 3:** Demographic characteristics at the initial examination.

	Patients with detected gastric cancer	Patients without detected gastric cancer	*P* value^a^
n	89	1342	
Examination period (months), mean (SD)	47.4 (32.8)	127.6 (4.1)	<0.001
Age (y), mean (SD)	56.7 (8.8)	46.2 (1.0)	<0.001
Sex (male), n (%)	75 (84.2)	1042 (77.6)	0.183
Body mass index (kg/m^2^), mean (SD)	23.3 (2.9)	23.1 (3.2)	0.539
*H. pylori*^b^ serology testing positive, n (%)	69 (77.5)	409 (30.4)	<0.001
**Upper gastrointestinal endoscopic findings**			
Chronic atrophic gastritis, n (%)	81 (91.0)	409 (30.4)	<0.001
Gastric or duodenal ulcers including scars, n (%)	21 (23.5)	118 (8.79)	<0.001
GERD^c^ or Barrett’s oesophagus, n (%)	20 (22.4)	312 (23.2)	0.969
Post-gastrectomy, n (%)	4 (4.49)	19 (1.41)	0.072
**Blood tests**			
White blood cell counts (×10^3^/μL), mean (SD)	5.866 (1.762)	5.510 (1.598)	0.0678
Neutrophil ratio (%), mean (SD)	59.1 (8.3)	57.3 (8.6)	0.0527
Lymphocyte ratio (%), mean (SD)	32.2 (7.1)	33.7 (8.0)	0.0591
Eosinophil ratio (%), mean (SD)	2.8 (1.8)	3.2 (2.6)	0.0278
Monocyte ratio (%), mean (SD)	5.5 (1.8)	5.3 (1.3)	0.275
Basophil ratio (%), mean (SD)	0.5 (0.3)	0.6 (0.4)	0.198
Haemoglobin (g/dL), mean (SD)	14.8 (1.0)	14.4 (1.3)	0.391
Mean corpuscular volume (fL), mean (SD)	94.4 (4.6)	92.3 (4.7)	<0.001
Platelet count (×10^4^/μL), mean (SD)	22.2 (4.8)	23.0 (5.1)	0.158
Haemoglobin A1c (%), mean (SD)	5.85 (0.87)	5.39 (0.56)	<0.001

^a^t-test or chi-squared test.

^b^*Helicobacter pylori, H. pylori*.

^c^Gastroesophageal reflux disease, GERD.

### XGBoost

XGBoost open source software^16,17^ provides machine learning solutions to classification and regression tasks using techniques of ensemble learning with gradient tree boosting (GTB)^41^. XGBoost is well known for obtaining winning solutions in various data competitions. Chen and Guestrin^16^ reported that “among the 29 challenge-winning solutions published on Kaggle’s blog during 2015, 17 winning solutions used XGBoost.” Practical applications of XGBoost include “store sales prediction, high energy physics event classification, Web text classification, customer behaviour prediction, motion detection, ad click-through rate prediction, malware classification, product categorization, hazard risk prediction and massive on-line course dropout rate prediction.” XGBoost has also been applied to the medical field^10–12^. We used XGBoost to classify subjects as high or low risk in terms of developing gastric cancer. XGBoost (or GTB) can capture nonlinear relations among the outcome and input variables by sequentially learning multiple CARTs; this jointly helps detect early signs of gastric cancer. CARTs possess parameters that need to be learned, such as various tree structure options and leaf node weights. XGBoost automatically learns multiple CARTs with these parameters by optimising a loss function using gradient methods. In addition, XGBoost includes several hyperparameters that need to be tuned, as described below.

### Evaluation

A trained classification model outputs a probability value [0–1] of the risk of developing gastric cancer for each participant. To evaluate the 10 trained classification models, we used ROC curves and their AUC values as computed from the test data^42,43^. ROC curves have commonly been used to measure classification performance. We report a confusion matrix (true positive, false negative, false positive, true negative) at the cut-off value determined by the probability value of 0.5 (i.e. if the probability value ≥0.5, then the patient is classified as being at risk; otherwise as low-risk).

### Hyperparameter tuning: bayesian optimization (BO)

XGBoost includes several hyperparameters that need to be tuned, including the maximum depth of regression trees, number of weak learners (CARTs), subsample ratios of columns and training instances for constructing each tree, partitioning-leaf-node parameters (gamma), imbalance parameters, learning rate (eta) and regularization parameters (lambda and alpha). The hyperparameter settings affect the performance of XGBoost. We optimized the hyperparameters to maximize the mean AUC value computed from 5-fold CV on the training data. Specifically, the training data were randomly divided into five subsets: four were used for training XGBoost and the other was used for validation. An ROC curve and AUC value could be evaluated from the validation subset. This procedure was repeated five times with different validation subsets, and then the mean AUC value was computed by averaging the five AUC values. We used the BO method^18,19^ to maximize automatically the mean AUC value (AUC-CV). BO iteratively suggests favourable hyperparameter values that effectively increase the objective function (AUC-CV) by learning the previously observed pairs of hyperparameters and their AUC-CV values. After identifying the favourable hyperparameter values, XGBoost was trained using the entire training dataset. The final ROC and AUC values were then evaluated using the test data (AUC-Test). Supplementary Figs S1 to S5 show the time courses of the monotonically increased AUC-CV values and resulting AUC-Test values for each model A–J as a function of iterations of BO explorations for hyperparameters. XGBoost and logistic regression showed a significant difference in models D, E, I and J with many input variables.

## Supplementary information

Supplementary Info

## Data Availability

The datasets generated during and/or analysed during the current study are not publicly available because of access to information being severely restricted by the ethics committee of NTT Medical Center Tokyo, but may be available from the corresponding author on reasonable request.
