# Supplementary material for: Prediction of future gastric cancer risk using a machine learning algorithm and comprehensive medical check-up data: A case-control study
Source: Sci Rep. 2019 Aug 27;9:12384. doi: 10.1038/s41598-019-48769-y (PMC6712020; doi:10.1038/s41598-019-48769-y)
Supplement: Supplementary file 1 — Supplementary Info [file 41598_2019_48769_MOESM1_ESM.pdf]

Title

Prediction of future gastric cancer risk using a machine learning algorithm and comprehensive medical check-up data: A case-control study

Authors

Junichi Taninaga[1], B.E., Yu Nishiyama[1], Ph.D., Kazutoshi Fujibayashi[2,4,5*], M.D, Ph.D., Toshiaki Gunji[3], M.D, Ph.D., Noriko Sasabe[3], M.D, Ph.D., Kimiko Iijima[3], M.D, Ph.D., Toshio Naito[2], M.D, Ph.D.

Affiliations

[1] Faculty of Informatics and Engineering, The University of Electro-Communications, Tokyo, Japan
[2] Department of General Medicine, School of Medicine, Juntendo University, Tokyo, Japan
[3] Center for Preventive Medicine, NTT Medical Center Tokyo, Tokyo, Japan
[4] Medical Technology Innovation Center, Juntendo University, Tokyo, Japan
[5] Clinical Research and Trial Center, Juntendo University Hospital, Tokyo, Japan

***Corresponding Author**

Kazutoshi Fujibayashi

Department of General Medicine, School of Medicine, Juntendo University

3-1-3, Hongo, Bunkyo-Ku, Tokyo 113-8421, Japan

Tel/Fax: 81(3)-5802-1190

E-mail: kfujiba@juntendo.ac.jp

Table S1. Results of predicting patients at risk of developing gastric cancer.

	TP[a]	FN[b]	FP[c]	TN[d]
Model A	12	3	86	186
Model B	15	0	103	169
Model C	13	2	87	185
Model D	15	0	68	204
Model E	14	1	63	209
Model F	0	15	0	272
Model G	0	15	0	272
Model H	15	0	105	167
Model I	0	15	2	270
Model J	9	6	27	245

[a] True Positive, TP

[b] False Negative, FN

[c] False Positive, FP

[d] True Negative, TN

Figure S1. Comparison of area under the curve (AUC) values between logistic regression and XGBoost in Models A and F.

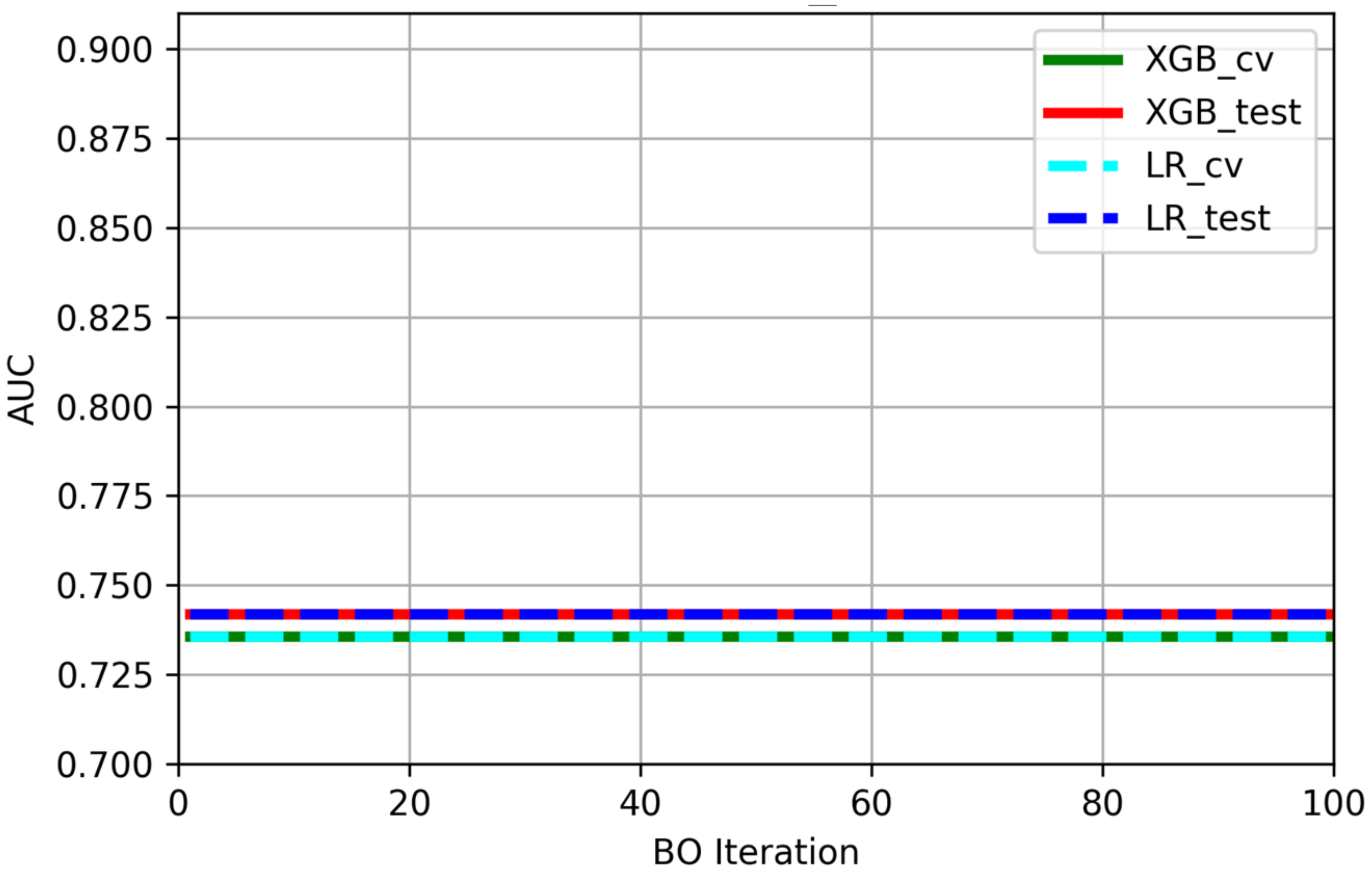

Bayesian optimisation (BO)

Figure S2. Comparison of area under the curve (AUC) values between logistic regression and XGBoost in Models B and G.

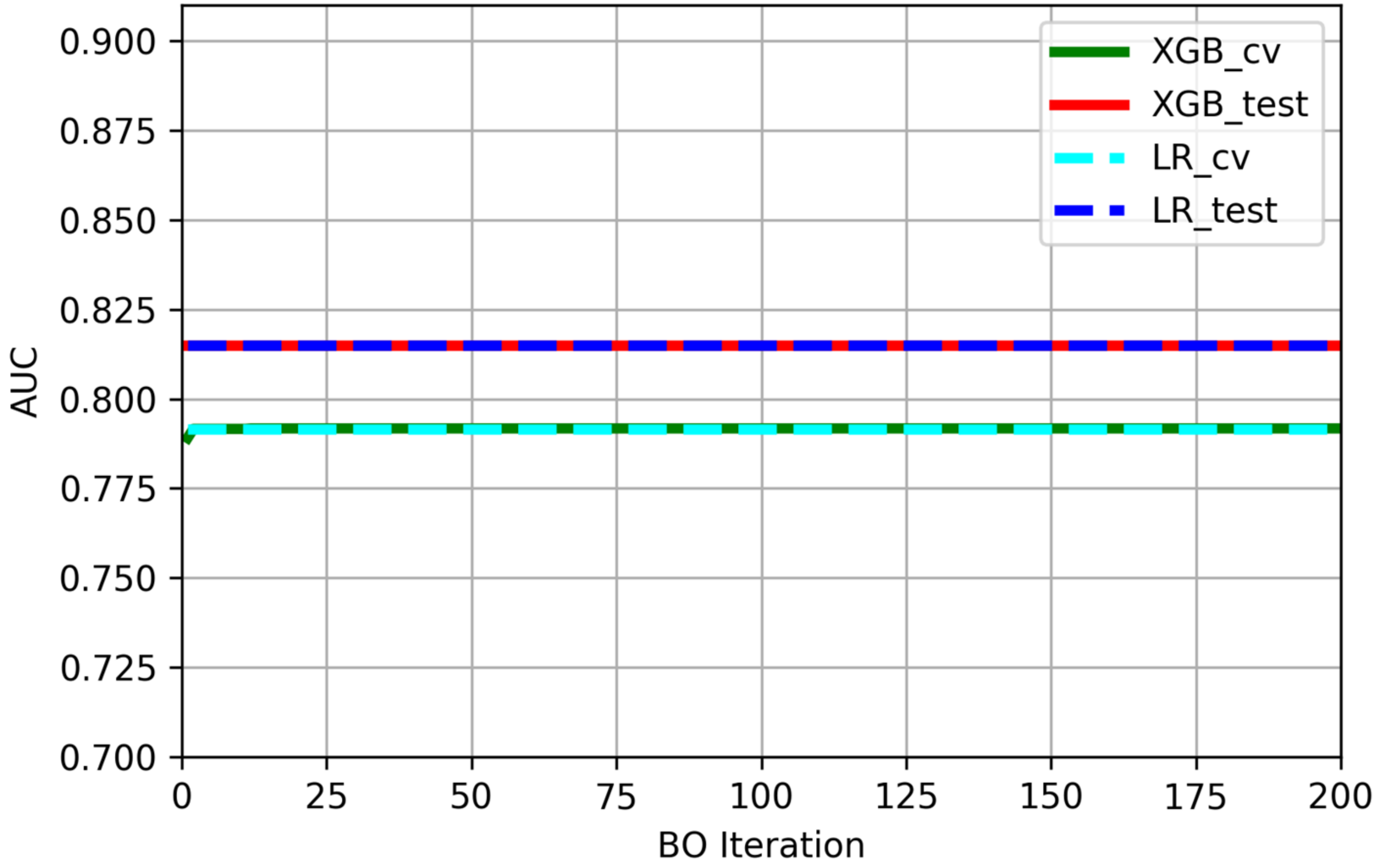

Bayesian optimisation (BO)

Figure S3. Comparison of area under the curve (AUC) values between logistic regression and XGBoost in Models C and H.

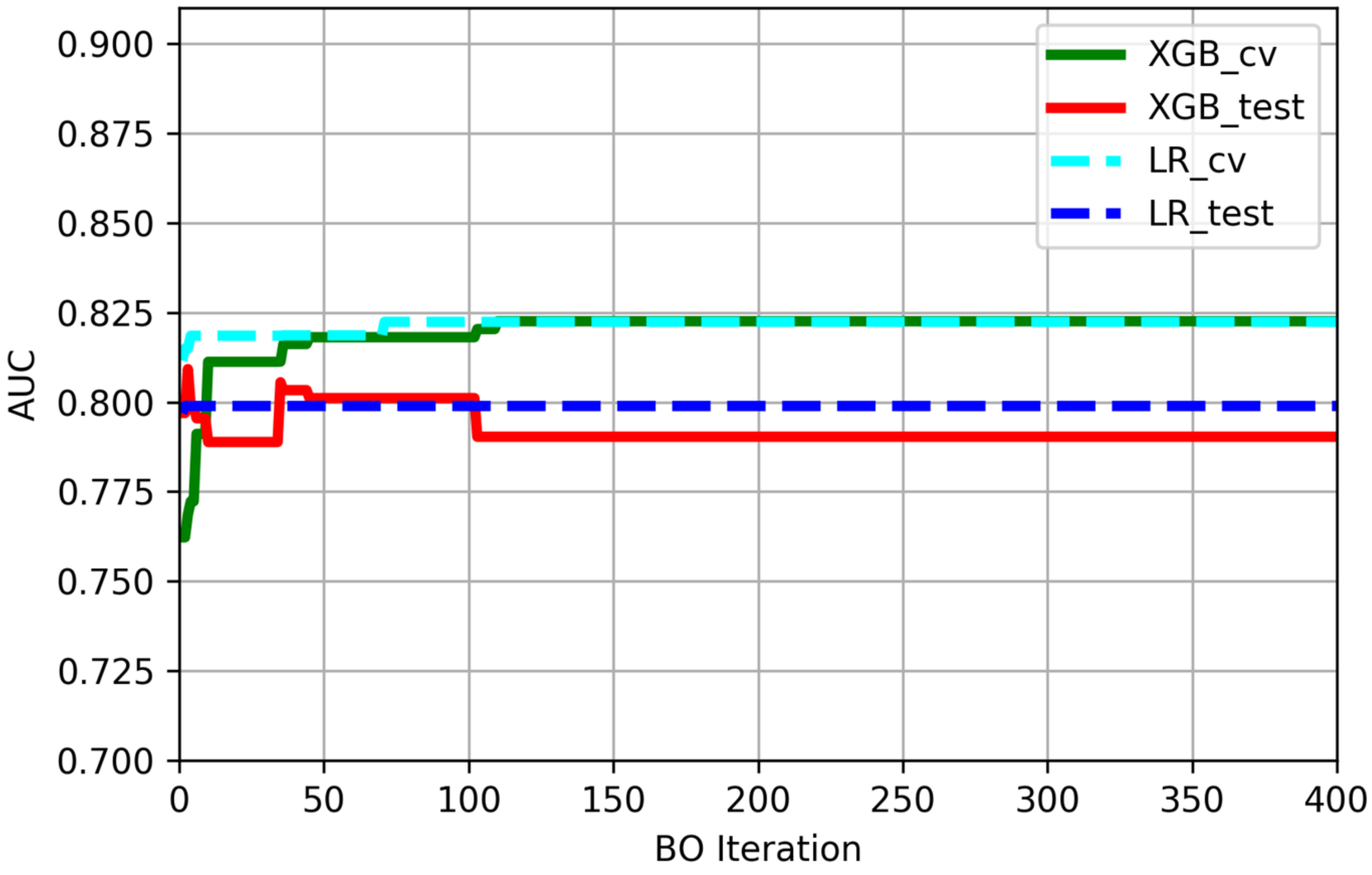

Bayesian optimisation (BO)

Figure S4. Comparison of area under the curve (AUC) values between logistic regression and XGBoost in Models D and I.

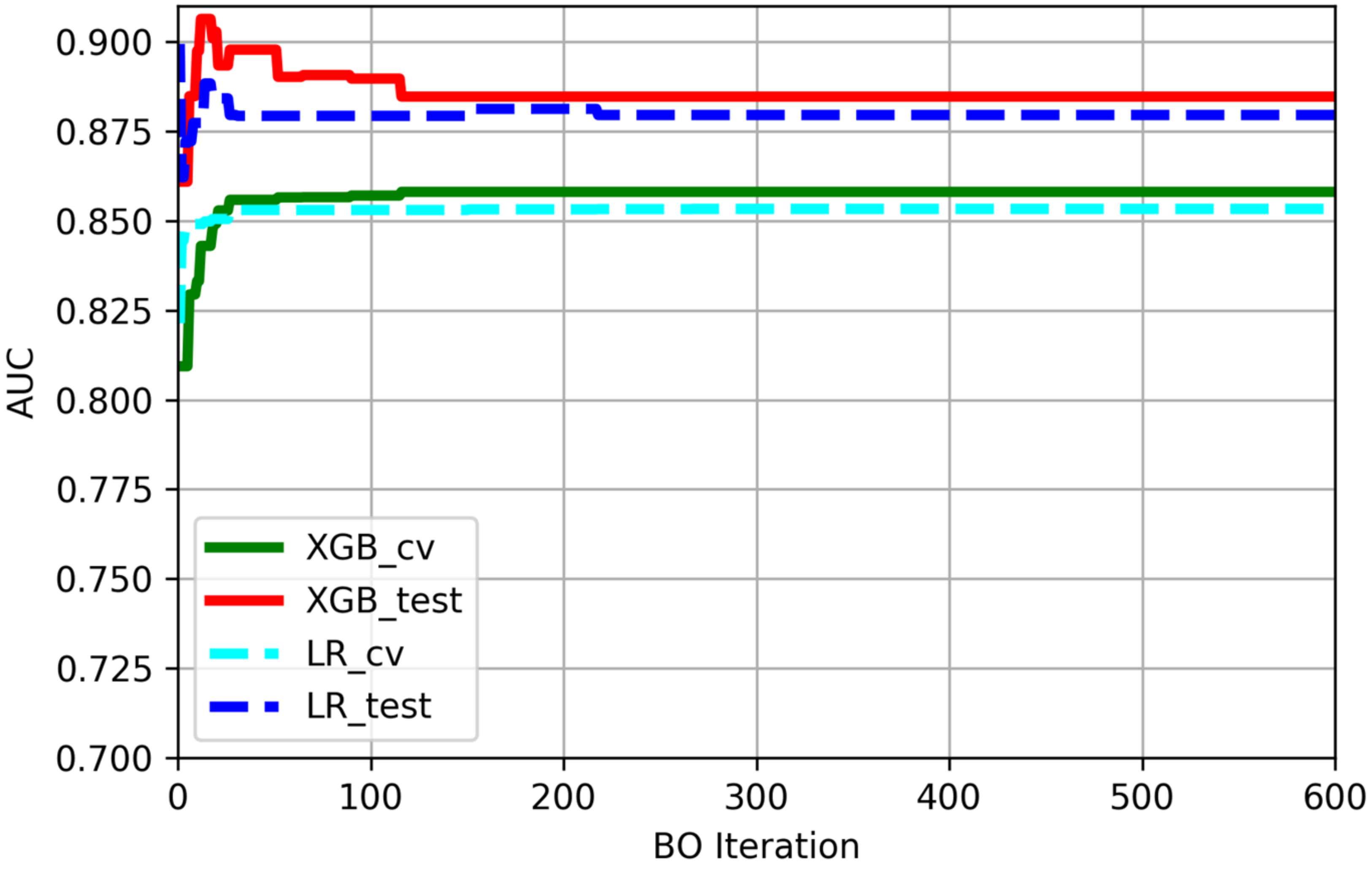

Bayesian optimisation (BO)

Figure S5. Comparison of area under the curve (AUC) values between logistic regression and XGBoost in Models E and J.

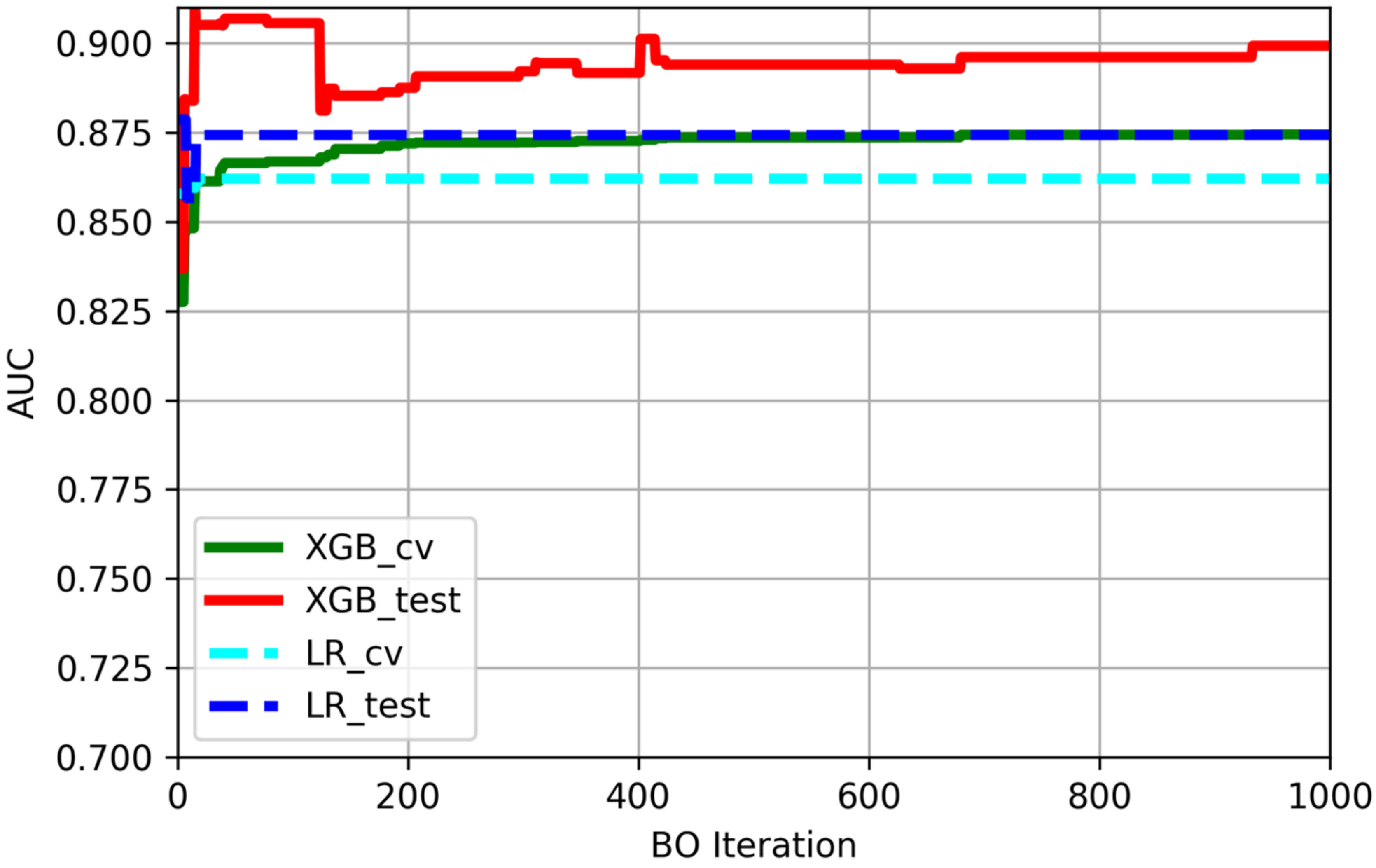

Bayesian optimisation (BO)

Table S2. Feature importance.

Model A	*H. pylori*[a] serology testing							
	1							
Model B	Chronic atrophic gastritis	*H. pylori* serology testing						
	0.647	0.353						
Model C	Chronic atrophic gastritis	Post-gastrectomy	Gastric or duodenal ulcers including scars	*H. pylori* serology testing	GERD[b] or Barrett's oesophagus			
	0.308	0.226	0.185	0.144	0.137			
Model D	Age	Chronic atrophic gastritis	*H. pylori* serology testing	Body mass index	Gastric or duodenal ulcers including scars	Post-gastrectomy	Sex	
	0.367	0.360	0.187	0.067	0.013	0.007	0.010	
Model E	Age	Mean corpuscular volume	Chronic atrophic gastritis	HbA1c	Lymphocyte ratio	*H. pylori* serology testing	Post-gastrectomy	Body mass index
	0.206	0.121	0.115	0.091	0.091	0.091	0.079	0.067

[a] *Helicobacter pylori, H. pylori*

[b] Gastroesophageal reflux disease, GERD

Table S3. Distribution of participants according to the number of upper gastrointestinal endoscopies.

Trials undergone<br>n	Patients with suspected gastric cancer<br>n	Patients without suspected gastric cancer<br>n
1	0	0
2	36	49
3	19	48
4	4	81
5	5	105
6	9	130
7	6	111
8	5	131
9	1	143
10	1	155
11	3	233
12	0	155
13	0	0
14	0	1
